# Position- and Time-Dependent *Arc* Expression Links Neuronal Activity to Synaptic Plasticity During Epileptogenesis

**DOI:** 10.3389/fncel.2018.00244

**Published:** 2018-08-14

**Authors:** Philipp Janz, Pascal Hauser, Katharina Heining, Sigrun Nestel, Matthias Kirsch, Ulrich Egert, Carola A. Haas

**Affiliations:** ^1^Experimental Epilepsy Research, Department of Neurosurgery, University Medical Center, University of Freiburg, Freiburg, Germany; ^2^Faculty of Biology, University of Freiburg, Freiburg, Germany; ^3^Laboratory for Biomicrotechnology, Department of Microsystems Engineering, University of Freiburg, Freiburg, Germany; ^4^Bernstein Center Freiburg, University of Freiburg, Freiburg, Germany; ^5^Institute for Anatomy and Cell Biology, Department of Neuroanatomy, University of Freiburg, Freiburg, Germany; ^6^Faculty of Medicine, University of Freiburg, Freiburg, Germany; ^7^BrainLinks-BrainTools Cluster of Excellence, University of Freiburg, Freiburg, Germany

**Keywords:** hippocampus, epilepsy, seizure, kainate, Arg3.1, spine

## Abstract

In mesial temporal lobe epilepsy (mTLE) an initial precipitating injury can trigger aberrant wiring of neuronal circuits causing seizure activity. While circuit reorganization is known to be largely activity-dependent, the interactions between neuronal activity and synaptic plasticity during the development of mTLE remain poorly understood. Therefore, the present study aimed at delineating the spatiotemporal relationship between epileptic activity, activity-dependent gene expression and synaptic plasticity during kainic acid-induced epileptogenesis in mice. We show that during epileptogenesis the sclerotic hippocampus differed from non-sclerotic regions by displaying a consistently lower power of paroxysmal discharges. However, the power of these discharges steadily increased during epileptogenesis. This increase was paralleled by the upregulation of the activity-related cytoskeleton protein (*Arc*) gene expression in dentate granule cells (DGCs) of the sclerotic hippocampus. Importantly, we found that *Arc* mRNA-upregulating DGCs exhibited increased spine densities and spine sizes, but at the same time decreased AMPA-type glutamate receptor (AMPAR) densities. Finally, we show that *in vivo* optogenetic stimulation of DGC synapses evoked robust seizure activity in epileptic mice, but failed to induce dendritic translocation of *Arc* mRNA as under healthy conditions, supporting the theory of a breakdown of the dentate gate in mTLE. We conclude that during epileptogenesis epileptic activity emerges early and persists in the whole hippocampus, however, only the sclerotic part shows modulation of discharge amplitudes accompanied by plasticity of DGCs. In this context, we identified *Arc* as a putative mediator between seizure activity and synaptic plasticity.

## Introduction

In mTLE, the most frequent form of intractable epilepsy in adults, seizures originate from the mesio-limbic network (Engel, 2001) and damage to the hippocampus is one of the leading causes for mTLE (Walker, 2015). The hippocampus is particularly vulnerable to *status epilepticus* (SE), head trauma or febrile seizures in childhood. Such initial precipitating insults can lead to the development of hippocampal sclerosis, characterized by the loss of pyramidal cells and hilar interneurons, and subsequent glial scarring. At the same time surviving DGCs disperse and establish recurrent synaptic connections in a process termed mossy fiber sprouting (Malmgren and Thom, 2012; Thom, 2014). It is generally assumed that the molecular, structural, and functional changes associated with hippocampal sclerosis develop during a latent seizure-free period which gradually primes the hippocampus to generate seizures. The latent period typically spans several years in human mTLE (Lukasiuk and Becker, 2014) and weeks to months in rodent models (Lévesque and Avoli, 2013). However, the definitive existence of this seizure-free, pre-epileptic state or the precise definition of its pathophysiological features is still under debate, since subclinical paroxysmal activity can emerge immediately after brain injury (Löscher et al., 2015). Thus, it is crucial to further delineate the series of pathological events that occur during epileptogenesis in order to understand why neuronal tissue becomes epileptic.

Recent studies suggested that in experimental mTLE the sclerotic hippocampus is involved in seizure generation and propagation (Pallud et al., 2011; Krook-Magnuson et al., 2013, 2015). Resection of sclerotic tissue in mesial temporal lobe regions is performed to achieve seizure control in human patients with intractable mTLE. However, a considerable fraction of patients does not remain seizure-free after surgery (Hennessy et al., 2000; Harroud et al., 2012), indicating that also more distant, non-sclerotic parts of the hippocampus or extra-hippocampal regions may contribute to seizure generation. In line with this notion, studies performed in acute slices prepared from epileptic mice, in which kainic acid (KA) had been injected into the hippocampus, revealed that particularly the non-sclerotic regions are prone to generate epileptiform activity, in contrast to sclerotic regions near the injection site (Le Duigou et al., 2008). Consistent with this finding, surviving DGCs in the sclerotic hippocampus downscale their intrinsic excitability (Young et al., 2009; Stegen et al., 2012; Kirchheim et al., 2013). Moreover, Häussler et al. (2012) showed that *in vivo* the amplitudes of paroxysmal discharges were lowest near the strongly sclerotic KA injection site and highest in the transition zone to non-sclerotic regions of the ipsilateral hippocampus. In fact, there is recent evidence that DGCs residing in the transition zone have an impaired gating function and thus fail to control the excitability of the whole hippocampus (Krook-Magnuson et al., 2015). Considering these controversial results, it is not clear whether the sclerotic hippocampus has indeed the potential to generate seizures, and how position-dependent alterations of the hippocampal network during early epileptogenesis relate to its pathological function in chronic mTLE.

To address these questions, we first determined the spatiotemporal properties of epileptiform activity, by comparing local field potential (LFP) recordings between sclerotic and non-sclerotic hippocampal sub-regions and at different time points during epileptogenesis. Then, we identified the corresponding active neuron populations and estimated their number of strong synaptic inputs by analyzing the extent and sub-cellular distribution of the *Arc* mRNA (also referred to as *Arg3.1*), which is expressed upon strong neuronal activity and transported to active synapses (Steward et al., 1998; Steward and Worley, 2001). Given that Arc holds a key role in regulating synaptic plasticity (Bramham et al., 2010; Korb and Finkbeiner, 2011; Shepherd and Bear, 2011), we also investigated alterations of postsynaptic spines on the structural and molecular level. Finally, we optogenetically stimulated the perforant path synapses on DGCs *in vivo*, to study the functional changes of the sclerotic hippocampal network upon synaptic input.

## Materials and Methods

### Animals

Experiments were conducted with adult (8–10 weeks) transgenic mice, in which eGFP is expressed under the control of the *Thy1* promoter (Feng et al., 2000). Mice were kept in a 12 h light/dark cycle at room temperature (RT) with food and water *ad libitum*. All animal procedures were carried out in accordance with the guidelines of the European Community’s Council Directive of 22 September 2010 (2010/63/EU) and were approved by the regional council.

### Kainate and Virus Injections

Mice were injected with KA into the right dorsal hippocampus, as described previously (Heinrich et al., 2006; Häussler et al., 2012; Janz et al., 2017a). Accordingly, mice were deeply anesthetized (ketamine hydrochloride 100 mg/kg, xylazine 5 mg/kg, atropine 0.1 mg/kg body weight, i.p.) followed by an stereotaxic injection of 50 nL of a 20 mM KA solution (Tocris, Bristol, United Kingdom) in 0.9% sterile saline. Stereotaxic coordinates were chosen relative to bregma: anterioposterior (AP) = -2.0 mm, mediolateral (ML) = -1.5 mm, and relative to the cortical surface: dorsoventral (DV) = -1.5 mm. Injections of 0.9% saline (50 nL) served as controls. Behavioral SE was verified by mild convulsive movements, chewing, rotations, or immobility, as described before (Riban et al., 2002; Heinrich et al., 2006). Only mice that experienced SE were considered for further analysis.

For optogenetic experiments 0.45 μl of an adeno-associated virus carrying the genomic sequences for channelrhodopsin 2 (ChR2) and mCherry under the control of the Ca^2+^/calmodulin-dependent kinase II alpha (CaMKIIa) promotor (AAV1.CaMKIIa.hChR2(H134R)-mCherry.WPRE.hGH; Penn Vector Core, PA, United States) was stereotaxically injected into the medial entorhinal cortex. Coordinates were chosen relative to bregma: AP = -5.0 mm, ML = -2.9 mm, and relative to the cortical surface: DV = -1.5 mm. Virus injection was performed in the same surgery as KA or saline injection (see above). To test for light-induced artifacts, Control mice (no-virus) did not receive any viral constructs.

### Electrode/Optic Fiber Implantation and Local Field Potential Recording

For LFP analysis, mice were implanted directly after KA injection with Teflon-coated platinum-iridium wires (125 μm diameter; World Precision Instruments, Sarasota, FL, United States) at three positions into the hippocampal formation: contralateral septal with coordinates relative to bregma: AP = -2.0 mm, ML = +1.4 mm, and relative to the cortical surface: DV = -1.7 mm; ipsilateral septal AP = -2.0 mm, ML = -1.4 mm, DV = -1.7 mm; and ipsilateral temporal AP = -3.4 mm, ML = -2.8 mm, DV = -2.1 mm. Two stainless steel screws (DIN 84; Schrauben-Jäger, Karlsruhe, Germany) were implanted above the frontal cortex as reference and ground electrodes, respectively. Electrodes were soldered to a micro-connector (BLR1-type, 25 poles; Bürklin, Oberhaching, Germany). In a parallel set of experiments, animals were additionally implanted with an optic fiber (ferrule 1.25 mm, cannula 200 μm diameter; Prizmatrix Ltd., Givat-Shmuel, Israel) in the ipsilateral septal hippocampus 3 weeks after KA injection. The implant was fixed with dental cement (Paladur; Kulzer, Hanau, Germany). Subsequently, mice were connected to a miniature preamplifier (Multi Channel Systems, Reutlingen, Germany). Signals were amplified 1000-fold, bandpass-filtered between 1 and 5 kHz and digitized with a sampling rate of 10 kHz (Power1401 analog-to-digital converter, Spike2 software; Cambridge Electronic Design, Cambridge, United Kingdom). In individual mice, LFP recordings were performed for at least 3 h at individual days post injection (dpi) (SE: 3 h post injection = 3 hpi; 1 week: 6–7 dpi; 2 weeks: 13–14 dpi; 3 weeks: 19–21 dpi).

### Analysis of Electrophysiological Data

LFP data was analyzed using a custom algorithm written in Python 2.7 (Python Software Foundation, Beaverton, OR, United States). In brief, we re-sampled the LFP to 500 Hz and calculated a spectrogram (fast Fourier transform) with a 128 point window. For recordings acquired during SE we used a shorter window (32 points) to resolve high-frequency discharge trains. We normalized the spectrogram in each frequency bin (dynamic normalization, Waldert et al., 2013). Next, we averaged these normalized frequencies in the 4–40 Hz range in each time bin and then z-scored across time. We defined peaks in this trace as epileptic spikes. This automatic spike detection procedure was validated by comparison to visual detections by an expert (average sensitivity: 0.87, average false positives/min: 1.9). A series of spikes was identified as a “burst” if at least five spikes occurred with an interval <1 s between one spike and the next. We further characterized each burst by its duration, spike rate and amplitude.

### Optogenetic Stimulation and Response Evaluation

Three weeks following KA injection, freely-behaving mice were optogenetically stimulated by delivering pulsed blue light (460 nm with about 60 mW/mm^2^ at the fiber tip; blue LED, Prizmatrix Ltd.) to ChR2-expressing entorhinal afferents in the ipsilateral septal hippocampus, while simultaneously LFPs were recorded (see above). To determine the seizure probability across different photo-stimulation frequencies, we used a stimulation protocol modified after Osawa et al. (2013): We applied 10 s long trains of pulsed blue light at different frequencies (1, 5, 10, or 20 Hz; duty ratio 0.33 or 0.1). For each frequency and duty ratio (the ratio between the light on- and off-time) we performed 10 trials, respectively. Individual trials were separated by 1 min and a minimum of 30 min in case a seizure was induced. Stimulation was started when the LFP signal had normalized (i.e., recovery of baseline amplitude and re-emergence of phases with theta rhythm). For each trial, post-stimulation effects (e.g., paroxysmal after-discharges) were manually evaluated on the electrophysiological and behavioral level. Full-blown seizures were identified electrophysiologically according to their generic characteristics described by Jirsa et al. (2014) and motor symptoms according to Racine (1972). The following categories were defined: (i) no response – no apparent changes, (ii) short paroxysmal burst – train of high-amplitude spikes lasting <10 s, (iii) paroxysmal episode – train of high-amplitude uniform spikes for >10 s without generic seizure characteristics according to Jirsa et al. (2014), (iv) short seizures – paroxysmal episode for <50 s with generic seizure characteristics and apparent behavioral manifestation according to Racine (1972), (v) long seizures – same as short seizures but lasting for <50 s. Example traces for a subclinical paroxysmal episode and a behavioral seizure are shown in **Figures 7A,B**, respectively.

### Perfusion and Tissue Preparation

KA-injected mice were anesthetized (see above) at 6 hpi, 7, 14, or 21 dpi and transcardially perfused with 0.9% saline followed by 4% paraformaldehyde in 0.1 M phosphate buffer (PB, pH 7.4) for 5 min. Controls were perfused either at 6 hpi or 21 dpi, since no apparent changes were observed between these two time points. Accordingly, controls for both time points were pooled. For ultrastructural analysis, 0.1% glutaraldehyde was added to the perfusion fixative. Following dissection, brains were post-fixated in the same fixative overnight, cryo-protected in sucrose (25% in PB-diethyl pyrocarbonate) over night at 4°C, shock-frozen in RNAse-free isopentane for 3 min at -40°C and stored at -80°C. Cryo-protection and subsequent freezing was left out for tissue used for electron microscopy. Brains were sectioned (50 μm, coronal plane) either with a cryostat (CM3050 Leica, Bensheim, Germany) for immunohistochemistry (IHC) and *in situ* hybridization (ISH), or on a vibratome (VT1000S, Leica) for electron microscopy. Slices were collected in 2× saline-sodium citrate buffer (2×SSC; 0.3 M NaCl, 0.03 M sodium citrate, pH 7.0) for ISH or in PB for IHC.

### *In situ* Hybridization

*Arc* mRNA was localized by ISH using digoxigenin (DIG)-labeled cRNA probes generated by *in vitro* transcription from an *Arc* encoding plasmid (Riken full length cDNA clone: B130064L16; Source BioScience, Nottingham, United Kingdom). In brief, the plasmids were linearized by restriction digest with *BamHI and NotI* to serve as template for T7 (sense) and T3 RNA polymerase (anti-sense). After *in vitro* transcription as previously described by Haas et al. (2000), DIG-labeled *Arc* cRNAs (3 kb) were purified by ethanol precipitation and were treated by alkaline hydrolysis to reduce their sizes to approximately 250 bases following standard protocols.

For ISH, cryo-sectioned brain slices were pre-treated in a 1:1 mixture of hybridization buffer [50% formamide, 4×SSC, 5% dextran sulfate, 250 μg/ml heat-denatured salmon sperm DNA, 200 μl yeast *t*-RNA, 1% Denhardt’s-reagent (Sigma-Aldrich, Steinheim, Germany)] and 2×SSC at RT for 15 min. Subsequently, the slices were pre-hybridized in hybridization buffer for 60 min at 55°C, followed by addition of DIG-labeled anti-sense or sense *Arc* cRNA probe (100 ng/ml) and incubated overnight at 55°C. Slices were washed in 2×SSC for 2 × 15 min at RT and then rinsed at 65°C for 15 min in 2×SSC with 50% formamide; 0.1 × SSC with 50% formamide and twice in 0.1 × SSC alone. Then the slices were rinsed in 0.1 M Tris-buffered saline (TBS) for 2 × 10 min and transferred to the blocking buffer [1% blocking reagent (Roche Diagnostics, Mannheim, Germany) in TBS] for 60 min at RT.

For subsequent immunohistological detection, the slices were treated with enzyme-coupled fab fragments against DIG. We used either colorimetric or fluorescent detection. For colorimetric detection sections were treated with alkaline phosphatase-coupled anti-DIG antibodies (1:1500; raised in sheep; Roche Diagnostics). The staining-reaction was performed in the dark using NBT/BCIP solution [3.4 μl nitroblue tetrazolium (100 mg/ml; Roche Diagnostics) and 3.5 μl 5-bromo-4-chloro-3-indolylphosphate (50 mg/ml; Roche Diagnostics) per 1 ml staining buffer: 100 mM Tris/HCl pH 9.5, 100 mM NaCl_2_, 50 mM MgCl_2_ in H_2_O] and slices were transferred into H_2_O when the desired intensity of the precipitate was reached (after about 1 h). The slices were mounted on glass slides, air dried and embedded in Kaisergelatine. ISH with sense probes proved the high specificity of our *Arc* mRNA detection (**Supplementary Figure 1**).

For fluorescent ISH (FISH) tissue sections were treated with a horseradish peroxidase-conjugated DIG antibody (1:2000, raised in sheep; Roche Diagnostics) developed in the presence of amplification buffer and tyramide working solution (1:50) for 6 min in the dark, using the Tyramide Signal Amplification (TSA) Plus Cyanine 3 System kit (PerkinElmer, Waltham, MA, United States). The staining-reaction was stopped by rinsing in TBS for 3 × 5 min and 1 × 15 min. Slices were kept in the dark for further fluorescent IHC.

### Immunohistochemistry

For immunofluorescence staining, free-floating coronal sections were pre-treated with 0.25% TritonX-100 (no Triton for FISH) in 1% bovine serum albumin (Sigma-Aldrich) in PB for 1 h. Subsequently, slices were incubated either with rabbit anti-GFP (1:1000; Abcam, Cambridge, United Kingdom), rabbit anti-mCherry (1:1000; Abcam) or rabbit anti-Arc (1:1000; Synaptic Systems, Göttingen, Germany) overnight at 4°C. For AMPAR staining, slices were pre-incubated with 0.2 mg/ml pepsin (Sigma-Aldrich) in 0.2 N HCl for 15 min at 37°C, washed with 20 mM TBS (pH 7.3) for 10 min and incubated with rabbit anti-AMPAR (1:500, Synaptic Systems, Göttingen, Germany) overnight at RT. Subsequently, slices were incubated with goat anti-rabbit Cy2, Cy3-, or Cy5-conjugated antibodies (1:200, Jackson ImmunoResearch Laboratories Inc., West Grove, PA, United States) for 2.5 h RT followed by rinsing in 0.1 M PB (or 20 mM TBS for AMPAR stained slices) for 6 × 15 min. Sections were mounted on uncoated glass slides and coverslipped for with ProLong Gold (Molecular Probes, Invitrogen, Carlsbad, CA, United States).

For electron microscopy, labeling of eGFP was carried out as described previously (Janz et al., 2017a). In brief, free-floating sections were pre-incubated with 20% normal goat serum in TBS for 1 h RT, followed by incubation with rabbit anti-GFP (1:5000; Abcam) at 4°C overnight. Then, sections were incubated with goat anti-rabbit 1.4 nm Nanogold antibody (1:100; Nanoprobes, Stony Brook, NY, United States) at 4°C overnight, enhanced with HQ silver kit (Nanoprobes) the next day, and contrasted using 0.5% OsO4 and 1% uranyl acetate. Following dehydratation and flat-embeddeding in Durcupan (Sigma-Aldrich), sections were trimmed and cut with a diamond knife.

### Image Acquisition and Analysis

Fluorescence and bright-field photomicrograph composites were taken with an AxioImager2 microscope using a Plan-APOCHROMAT 10× objective (Zeiss, Göttingen, Germany). Exposure times (NBT/BCIP-labeled *Arc* mRNA: 2 ms; Cy3-labeled *Arc* mRNA: 1 s; Cy5-labeled AMPAR protein: 4.5 s) were kept constant for each staining to allow for comparisons across individual sections. The images were further processed with ZEN blue software (Carl Zeiss, Göttingen, Germany). The optical density of AMPAR labeling was assessed using conventional epifluorescence microscopy images, whereas for *Arc* FISH measurements we used images from confocal laser-scanning microscopy (see paragraph below). For each animal, AMPAR densitometry was determined in the molecular layer of three similar sections of the ipsilateral septal and the contralateral septal hippocampus. Using the Fiji ImageJ software (Schindelin et al., 2012), the images were converted to gray-scale, a region-of-interest (ROI) was drawn comprising the supra- and infra-pyramidal part of the molecular layer using the “polygon” function, and the mean intensity was calculated. The upper and lower boarders of the molecular layer were defined by the upper end of the granule cell layer and the hippocampal fissure, respectively.

**FIGURE 1:**
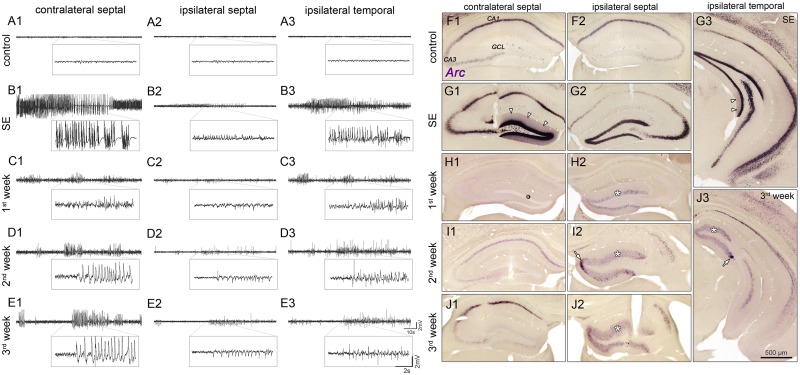
Spatiotemporal patterns of hippocampal activity during the development of mTLE. **(A)** Representative LFP recordings from three regions in the hippocampus of a saline-injected control mouse (1, contralateral septal; 2, ipsilateral septal; and 3, ipsilateral temporal) and **(B–E)** of one epileptic mouse at different time points (**B**, SE; **C**, first week; **D**, second week; **E**, third week) following KA injection. Note, that epileptiform activity is present throughout epileptogenesis, exhibiting position-dependent differences in power of paroxysmal discharges: Low amplitudes in the sclerotic (ipsi septal) hippocampus; high amplitudes in non-sclerotic regions (contra septal; ipsi temporal). **(F–J)** Corresponding time course of *Arc* mRNA expression. CA1/3, cornu ammonis 1 and 3; GCL, granule cell layer. Arrow heads in **G1** and **G3** denote dendritic translocation during SE. Arrows in **I2** and **J3** mark increased *Arc* expression near the transition to the non-dispersed GCL in chronic epilepsy. Note that in the ipsilateral GCL *Arc* expression persists for 3 weeks during epileptogenesis (asterisks).

In order to assess the density of *Arc* FISH signals in the somata of DGCs, three confocal images were taken per region and animal (contralateral septal, ipsilateral septal, ipsilateral temporal hippocampus – near the respective electrode position), with an Olympus FV10i confocal laser-scanning microscope (Olympus Deutschland, Hamburg, Germany) using a 10× objective. Image resolution was 1024 × 1024 pixels. Each image was averaged four times. Confocal aperture was set to 1.0. For excitation of Cy3-labeled *Arc* mRNA the power of the laser diode (559 nm) was set to 10%. For detection of the fluorescence signal the sensitivity of the photomultiplier was set to 50%. Acquired images were transferred to Fiji Image J, converted to gray-scale, and a ROI was drawn around the granule cell layer of the supra- and infra-pyramidal blade. For the ipsilateral temporal region only the ventral half of the granule cell layer was selected, since the dorsal half displayed sclerosis similar to the ipsilateral septal region (see **Figures 1J3, 3D3**). For quantitative analysis the integrated optical density of the *Arc* FISH signal was calculated to compensate for epilepsy-induced dispersion of DGCs. We chose this rather crude quantification method since in the granule cell layer the *Arc* FISH signal was too dense for single-profile counts.

Conversely, we were able to detect individual Cy3-labeled *Arc* FISH profiles in the molecular layer due to their sparse distribution. Thus, for each animal two to four confocal *z*-stacks (step size 0.5 μm; image resolution 1024 × 1024 pixels) per region (see above) were acquired with an FV10i laser-scanning microscope (Olympus Deutschland, Hamburg, Germany) using a 60× oil-immersion objective. Each image was averaged four times. Confocal aperture was set to 1.5. For excitation of Cy3 the power of the laser diode (559 nm) was set to 15% and for detection the sensitivity of the photomultiplier was set to 50%. Deconvolution of confocal *z*-stacks was performed with Huygens Core (Scientific Volume Imaging B.V., Hilversum, Netherlands). Image post-processing and analysis was performed with Imaris 7.7.1 software (Bitplane AG, Zurich, Switzerland). The intensity along the *z*-axis was normalized and the background threshold was set to five (of 255). A Gaussian filter (width: 0.14 μm) was applied for smoothing. Using the “Add new spots” function, individual Cy3-labeled *Arc* FISH profiles were automatically detected (specific parameters can be found in **Supplementary Figure 2**). Quantification was done in three predefined volumes (40 × 40 × 20 μm^3^) per region (middle and outer molecular layer: defined by the middle third and the outer third of the layer, respectively). We choose three separate volumes to account for curving of the molecular layer. For these volumes, the calculated densities of *Arc* FISH profiles were averaged.

For the analysis of GFP-positive DGC spines, image acquisition was performed as described in the former paragraph, with the exception that we used a laser diode setting (473 nm; 10% laser power) for excitation of GFP. Photomultiplier sensitivity was set to 40%, 15–30 separate, clearly visible dendritic segments (mean ± SD length: 43.9 ± 19.5 μm), crossing the middle or outer molecular layer, were selected per animal and analyzed by a blinded observer using Fiji Image J. Spines were identified as protrusions on individual GFP-labeled dendrites and counted with the “multi-point” function. The length of the corresponding dendritic segment was measured with the “segmented line” function. Resulting spine counts were normalized on the dendrite length to calculate the number of spines per μm.

The ultrastructure of dendritic spines was investigated in transmission electron microscopy images. For each animal, 15–25 randomly selected images were taken in the molecular layer, using a LEO 906E microscope (Zeiss), and analyzed with ImageSP software (Sysprog, Minsk, Belarus). Spines were identified by a clearly visible postsynaptic density (PSD), the presence of an adjacent presynaptic bouton and a preserved connection of the spine head with the dendritic shaft. For each spine the perimeter as well as the length and the number of PSDs per spine were measured, using the “polygon” or “segmented line” function, respectively.

### Statistical Analysis

Data was tested for significant differences with Prism 5 software (GraphPad Software Inc., La Jolla, CA, United States). Comparisons versus controls were performed with an unpaired Student’s *t*-test. When more than two groups were compared a one-way ANOVA followed by Bonferroni’s post-test was used. Significance thresholds were set to: ^∗^*p* < 0.05, ^∗∗^*p* < 0.01, ^∗∗∗^*p* < 0.001. For all values, mean and standard error of the mean (SEM) are given. Correlations were tested using Pearson’s correlation (slope significantly non-zero, confidence interval set to 95%).

## Results

### Early Appearance and Persistence of Paroxysmal Discharges in the Hippocampal Network During Epileptogenesis

First, we performed LFP recordings over time at three positions in the hippocampus (contralateral septal, ipsilateral septal and ipsilateral temporal) of KA- and saline-injected animals (**Figures 1A1–E3**), in order to describe position-dependent activity dynamics during the development of mTLE. Active cell populations were identified by *post-hoc* ISH for *Arc* mRNA in the same hippocampal regions (**Figures 1F1–J3**).

Under control conditions, no paroxysmal discharges (i.e., epileptic bursts, as defined in “Material and Methods”) were evident (**Figures 1A1–A3**) and *Arc* mRNA was mainly expressed in CA1 pyramidal cell somata, to a lesser extent in CA2/3 pyramidal cells and very sparsely in DGCs. During SE (3–6 h following KA injection into the septal hippocampus) prolonged seizures emerged bilaterally, displaying substantially higher amplitudes in the contralateral septal and ipsilateral temporal regions compared to the injection site (**Figures 1B1–B3**). At this time point, somatic *Arc* mRNA levels were strongly upregulated bilaterally in DGCs, pyramidal cells and hilar interneurons, but translocation into the dendritic compartment was only apparent in DGCs of the non-sclerotic hippocampus (contralateral and ipsilateral temporal; **Figures 1G1–G3**). The level of *Arc* mRNA apparently correlated with the seizure amplitudes, i.e., being higher in the non-sclerotic regions of the hippocampus. During subsequent epileptogenesis both, seizure activity and *Arc* expression declined strongly. However, low-amplitude paroxysmal bursts that appeared synchronously in the hippocampal formation persisted during 3 weeks of epileptogenesis (**Figures 1C1–E3**). Note, that the vast majority of epileptic bursts were sub-clinical, meaning that they were not associated with behavioral changes according to Racine (1972). During this period, the position-dependent amplitude differences were still apparent, but to our surprise the *Arc* mRNA expression pattern was inverted, exhibiting higher levels in ipsilateral septal DGCs (**Figures 1H1–J3**). In fact, *Arc* mRNA expression in the dentate gyrus was highly restricted to dispersed DGCs in the chronic stage of mTLE (**Figures 1J1–J3**). Beside DGCs, remaining pyramidal cells also showed considerable *Arc* mRNA expression, indicating their functional integration in the epileptic hippocampal network.

**FIGURE 2:**
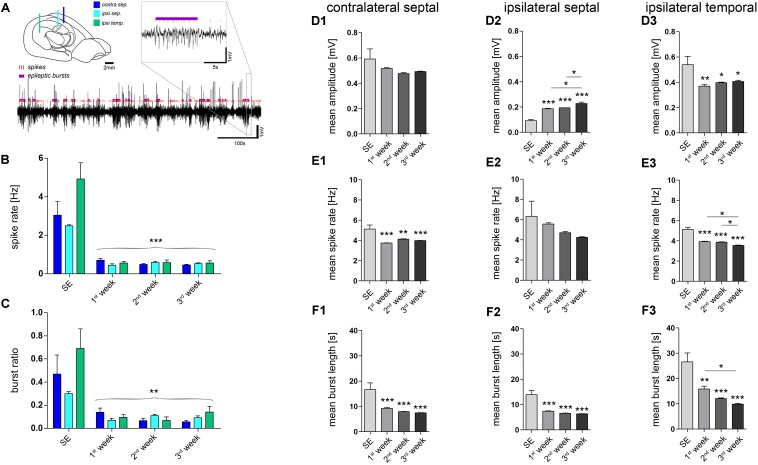
Quantitative analysis of paroxysmal burst features. **(A)** Schematic illustrating the recording sites and a representative LFP trace in which individual population spikes (red lines) and paroxysmal episodes (violet bars) were detected by an automated algorithm. Brain sketch modified from Witter and Amaral (2004). **(B)** Quantitative analysis of the overall spike rate per recording and **(C)** the relative time spent in a paroxysmal state (burst ratio). Color-code corresponds to legend in A. **(D–F)** Quantitative analysis of burst features for each hippocampal location: **(D1–D3)** Mean amplitude during burst; **(E1–E3)** mean spike rate; **(F1–F3)** mean burst length (1, contralateral septal; 2, ipsilateral septal; and 3, ipsilateral temporal). Number of mice: *n*_SE_ = 3, *n*_1w_ = 4, *n*_2w_ = 3, *n*_3w_ = 5. One-way ANOVA, Newman–Keuls’s post-test. ^∗^*P* < 0.05, ^∗∗^*P* < 0.01, ^∗∗∗^*P* < 0.001. Significance signs above bars indicate significant difference to SE values. All values are represented as the mean+SEM.

Quantitative analysis of epileptiform events (**Figure 2**) at different time points during epileptogenesis (*n*_cntrl_ = 4; *n*_SE_ = 3, *n*_1w_ = 4, *n*_2w_ = 3, *n*_3w_ = 5) corroborated our observations. First, we determined the number of putatively epileptic spikes per recording (spike rate) and the time spent in a paroxysmal state in relation to seizure-free episodes (burst ratio). Apparently, control mice showed no spontaneous epileptiform activity. Conversely, upon KA injection all mice displayed clearly identifiable epileptic episodes that showed modulation of amplitude, duration and spike frequency over time. During SE we observed prolonged seizure activity lasting for about 6 h. At 1 week after SE, we found a pronounced decrease of epileptic spikes and paroxysmal bursts to about 10–20% of SE values, which remained largely constant during the subsequent 2 weeks (**Figures 2B,C**). Interestingly, although paroxysmal episodes were actually present on both sides of the hippocampus and often appeared synchronously, the burst ratio for the contralateral side was slightly decreased during 3 weeks of epileptogenesis (SE: 0.468 ± 0.166; 1w: 0.140 ± 0.035; 2w: 0.066 ± 0.022; 3w: 0.056 ± 0.013). Conversely, the burst ratio calculated for both ipsilateral regions appeared more constant (ipsi sept, SE: 0.299 ± 0.018; 1w: 0.070 ± 0.017; 2w: 0.110 ± 0.012; 3w: 0.092 ± 0.015; ipsi temp, SE: 0.690 ± 0.170; 1w: 0.095 ± 0.022; 2w: 0.069 ± 0.030; 3w: 0.140 ± 0.050).

Next, we investigated whether the detected paroxysmal bursts change their characteristics during the course of epileptogenesis. The mean amplitude of spikes during paroxysmal bursts increased gradually over time only in the ipsilateral septal hippocampus (**Figure 2D2**; SE: 0.092 ± 0.009 mV; 1w: 0.186 ± 0.004 mV; 2w: 0.193 ± 0.001 mV; 3w: 0.228 ± 0.011 mV; refer to figure for *P* values). Conversely, the mean spike rate within a burst and its mean duration showed a trend to decrease (**Figures 2E2, F2**; spike rate, SE: 6.33 ± 1.49 Hz; 1w: 5.56 ± 0.13 Hz; 2w: 4.71 ± 0.11 Hz; 3w: 4.24 ± 0.06 Hz; burst length, SE: 14.0 ± 1.66 s; 1w: 7.4 ± 0.2 s; 2w: 6.6 ± 0.1 s; 3w: 6.4 ± 0.1 s; refer to figure for *P* values). In turn, the same course was statistically significant in the more temporal part of the ipsilateral hippocampus (**Figures 2E3, F3**; spike rate, SE: 5.12 ± 0.22 Hz; 1w: 3.96 ± 0.05 Hz; 2w: 3.86 ± 0.04 Hz; 3w: 3.55 ± 0.04 Hz; burst length, SE: 26.6 ± 3.5 s; 1w: 15.9 ± 1.1 s; 2w: 12.1 ± 0.3 s; 3w: 9.9 ± 0.3 s; refer to figure for *P* values), moreover showing a trend of increased burst amplitudes similar to the septal region (**Figure 2D3**; SE: 0.540 ± 0.065 mV; 1w: 0.370 ± 0.012 mV; 2w: 0.398 ± 0.005; 3w: 0.407 ± 0.009 mV). We also analyzed the line length (voltage change per burst duration) of individual epileptic bursts to give an estimate for their severity, revealing an increase during 3 weeks of epileptogenesis in the sclerotic ipsilateral septal hippocampus (**Supplementary Figure 3**).

Taken together, our data show that on both sides of the hippocampus paroxysmal bursts occur early after SE and persist throughout epileptogenesis. Beside moderate modulation of the spike rate and burst duration, the power of paroxysmal bursts exhibit position- and time-dependent differences: (i) Higher amplitudes in non-sclerotic versus sclerotic parts. (ii) Progressive increase of amplitudes only in the sclerotic hippocampus. *Arc* mRNA detection, as a surrogate marker for active neurons, indicates that epileptic activity in non-sclerotic regions may encompass mainly pyramidal cells, whereas in sclerotic regions primarily hyperactive DGCs and remaining pyramidal cells are involved.

### Position-Dependent Activity Changes During Epileptogenesis Determine the Sub-cellular Distribution of Arc mRNA Expression

**FIGURE 3:**
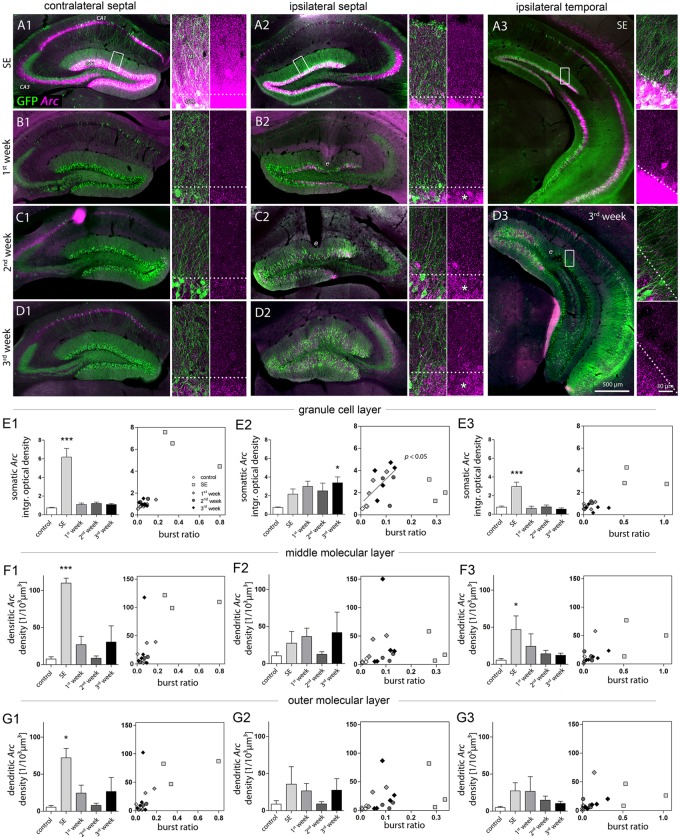
Characterization of DGC activity and synaptic input based on *Arc* expression. **(A–D)** Representative images showing *Arc* mRNA expression detected by FISH (magenta) during epileptogenesis (**A**, SE; **B**, first week; **C**, second week; **D**, third week). Transgenic Thy1-eGFP mice were used in which a subset of DGCs and also some pyramidal cells are intrinsically labeled with GFP (green). Each panel shows an overview of the hippocampus (left) and high-resolution confocal images of the dentate gyrus (right; displayed as a 3D maximum projection). Dashed lines denote the border between the granule cell layer (GCL) and the molecular layer (ML). Following SE, *Arc* mRNA stays upregulated in the ipsilateral GCL (asterisks). *e* indicates tissue distorted by electrodes. **(E–G)** Quantitative analysis of *Arc* mRNA density (left panel) in different layers (**E**, granule cell layer; **F**, middle and **G**, outer molecular layer) at different hippocampal locations (1, contralateral septal; 2, ipsilateral septal; and 3, ipsilateral temporal). Number of mice: *n*_cntrl_ = 3–5, *n*_SE_ = 3, *n*_1w_ = 3, *n*_2w_ = 3, *n*_3w_ = 5. One-way ANOVA, Newman–Keuls’s post-test. ^∗^*P* < 0.05, ^∗∗∗^*P* < 0.001. Right panels show the corresponding linear-regression analysis (Pearson’s correlation) between *Arc* mRNA and the burst ratio. Correlations were performed excluding SE values (gray regression lines and *P* values), considering SE as a special case due to acute effects of KA on the network activity.

DGCs have a key role in gating afferent input to the hippocampus (Hsu, 2007; Krook-Magnuson et al., 2015) and thus, are critically involved in regulating local network activity. Therefore, we characterized the spatiotemporal activity alterations of DGCs during epileptogenesis in more detail using *Arc* mRNA as a molecular marker for strong neuronal or synaptic activity. We did so by applying a FISH protocol, which allowed us to infer the *Arc* mRNA distribution at a sub-cellular resolution (**Supplementary Figure 4**), and to determine not only the overall expression level but also its transport to highly-active synapses in relationship to local seizure activity (**Figure 3**). As before, we analyzed “steady state” *Arc* mRNA levels at several time points (*n*_cntrl_ = 5; *n*_SE_ = 3, *n*_1w_ = 3, *n*_2w_ = 3, *n*_3w_ = 5) to estimate the time course of DGC activation during epileptogenesis. Here, we chose to measure the integrated optical density of *Arc* mRNA labeling in the granule cell layer for somatic expression, in contrast to the number of labeled profiles in the molecular layer for dendritic expression, since the somatic labeling was too dense to identify individual profiles.

Qualitative evaluation of FISH-based *Arc* mRNA detection confirmed, that during SE the upregulation of *Arc* expression in DGCs directly reflected the amplitude of local paroxysmal activity which was higher in regions not directly affected by KA (for *Arc* expression under control conditions, see **Supplementary Figure 4**). Accordingly, in contralateral septal and ipsilateral temporal DGCs the rise in somatic *Arc* mRNA expression during SE was considerably stronger (**Figures 3A1–A3, E1–E3**; optical density; contralateral septal: 6.18 ± 0.92; ipsilateral septal: 2.16 ± 0.57; ipsilateral temporal: 3.00 ± 0.47) and *Arc* mRNA also translocated to dendritic compartments in the molecular layer (**Figures 3F1–F3**; number of profiles; contra sept: 110.0 ± 6.56/10^3^ μm^3^; ipsi sept: 27.59 ± 15.70/10^3^ μm^3^; ipsi temp: 46.72 ± 18.45/10^3^ μm^3^). In these non-sclerotic regions the increase of *Arc* mRNA was only transient, showing control values at 1 week following SE. Conversely, in ipsilateral septal DGCs the SE-induced *Arc* mRNA upregulation stayed elevated during 3 weeks of disease progression, reaching significance after 3 weeks of epileptogenesis (**Figures 3A2, B2, C2, D2, E2**; control: 0.73 ± 0.06; SE: 2.16 ± 0.57; 1w: 2.99 ± 0.57; 2w: 2.51 ± 0.85; 3w: 3.39 ± 0.63, *P* < 0.05). The same spatiotemporal pattern of *Arc* mRNA expression was evident, when the optical density values were normalized to the intensity of the hilus, showing that variation of the background does not explain our results (**Supplementary Figure 5**). No clear translocation of *Arc* mRNA to the dendritic compartment was evident, which was reflected by the large variability of the number of *Arc* mRNA profiles in the molecular layer (**Figure 3F2**; control: 10.55 ± 4.96/10^3^ μm^3^; SE: 27.59 ± 15.70/10^3^ μm^3^; 1w: 36.54 ± 11.29/10^3^ μm^3^; 2w: 12.42 ± 3.6/10^3^ μm^3^; 3w: 42.0 ± 27.44/10^3^ μm^3^). Linear regression analysis for somatic *Arc* mRNA expression and epileptic activity showed that both measures were positively correlated for ipsilateral septal DGCs (**Figure 3E2**; R^2^ = 0.42, *P* < 0.05). For this analysis the SE time point was excluded, due to acute effects and direct actions of KA on activity-dependent gene expression.

**FIGURE 4:**
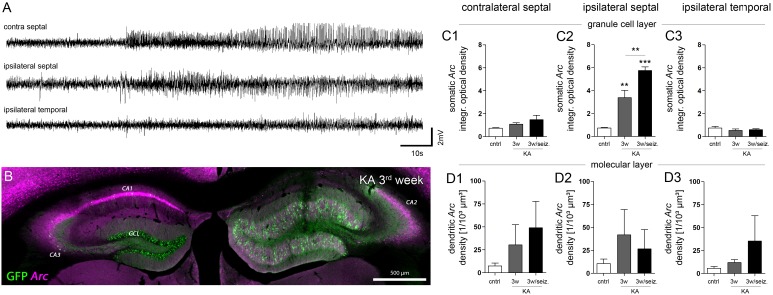
Effect of paroxysmal episodes on *Arc* mRNA expression and translocation. **(A)** Representative LFP recording of a long (>30 s) paroxysmal episode 3 weeks after KA injection. **(B)** Representative image of *Arc* expression (magenta) detected 30 min after the long paroxysmal episode shown in **A**. **(C)** Quantitative analysis of *Arc* mRNA in DGCs somata and **(D)** dendrites at different hippocampal locations (1, contralateral septal; 2, ipsilateral septal; and 3, ipsilateral temporal). Chronically epileptic mice which experienced at least one long paroxysmal discharge within 30 min before perfusion (*n*_3w/seiz_ = 4), were compared to chronically epileptic mice without any long paroxysmal discharge within 30 min before perfusion, or were compared to healthy controls (*n*_3w_ = 5; *n*_cntrl_ = 5; note that this data is also presented in **Figure 3**). One-way ANOVA, Newman–Keuls’s post-test; ^∗∗^*P* < 0.01, ^∗∗∗^*P* < 0.001.

Next, we investigated whether the prevailing unilateral *Arc* mRNA upregulation in DGCs was actually regulated by activity or just a molecular peculiarity of these cells directly affected by KA (**Figure 4**). We chose an experimental design in which we recorded LFPs from a subset of mice (*n* = 4) in the chronic stage of epilepsy (3 weeks following SE), then we identified the first long (>30 s) paroxysmal episode in a given recording session (**Figure 4A**), and sacrificed the animal 30 min later approximately at the peak of *Arc* mRNA expression kinetics. In fact, compared to epileptic mice which did not exhibit a long paroxysmal episode (30 min before sacrifice), we found an additional upregulation of somatic, but not of dendritic *Arc* mRNA in DGCs of the sclerotic region (**Figures 4B, C1–C3, D1–D3**; granule cell layer, 3w: 3.386 ± 0.630; 3w/seizure: 5.755 ± 0.310, *P* < 0.01; middle molecular layer, 3w: 41.96 ± 27.44/10^3^ μm^3^, 3w/seizure: 26.63 ± 21.14/10^3^ μm^3^). Considering the rapid transport of *Arc* mRNA into dendrites (>10 μm/min, max. speed of 60 μm/min; Dynes and Steward, 2007), we would have expected to detect dendritic translocation in DGCs – if there is – within minutes during the peak of somatic *Arc* expression (i.e., 30 min after seizure). However, we cannot exclude that dendritic translocation in DGCs might be slower or impaired under epileptic conditions, which could also explain a lack of *Arc* mRNA upregulation in the molecular layer. Beyond DGCs, activity-dependent expression of *Arc* mRNA was also induced in remaining, presumably CA2 pyramidal cells on the ipsilateral side (according to Häussler et al., 2016) and CA1-2 pyramidal cells on the contralateral side (**Figure 4B**), indicating that these cells are active components during paroxysmal episodes as well.

### Epilepsy-Associated Structural and Molecular Plasticity of DGC Synapses

**FIGURE 5:**
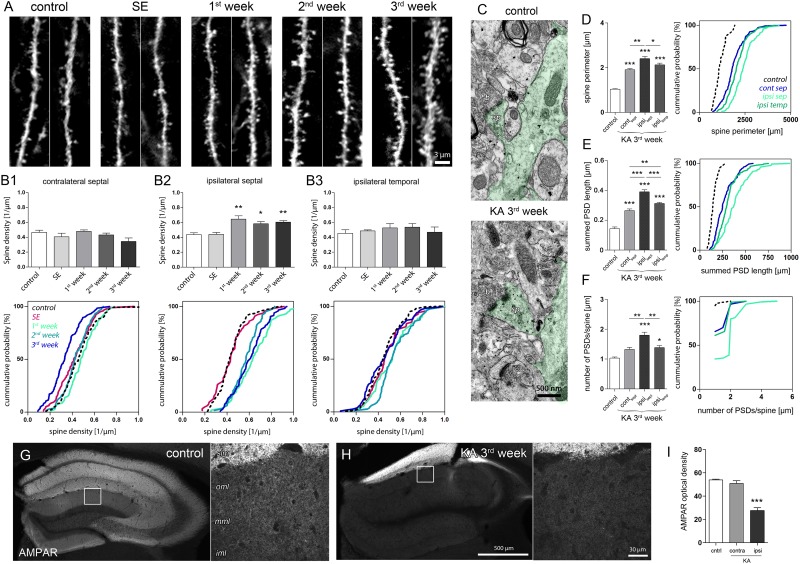
Structural and molecular site plasticity of excitatory postsynaptic contacts on DGCs. **(A)** Representative confocal images of individual eGFP-labeled DGC dendritic segments located in the middle or outer molecular layer of the ipsilateral septal hippocampus. Two dendritic segments are shown for each time point. **(B1–B3)** Quantitative analysis of DGC spine density changes during epileptogensis. Number of mice: *n*_cntrl_ = 5, *n*_SE_ = 3, *n*_1w_ = 4, *n*_2w_ = 3, *n*_3w_ = 5. One-way ANOVA, Newman–Keul’s post-test. ^∗^*P* < 0.05, ^∗∗^*P* < 0.01, ^∗∗∗^*P* < 0.001. Corresponding cumulative probability plots are shown below. **(C)** Representative electron microscopy images of an eGFP-labeled dendrite (green shading) in the middle molecular layer. sp, spine; b, bouton. **(D–F)** Quantitative analysis of ultrastructural spine features (perimeter; summed length of PSD, number of PSDs per spine) in controls and chronically epileptic mice. Ultrastructural features are compared between different hippocampal locations (contralateral septal: *n*_contra-sept_ = 5; ipsilateral septal: *n*_ipsi-sept_ = 6, ipsilateral temporal: *n*_ipsi-temp_ = 6) and control hippocampi (ipsilateral septal: *n*_cntrl_ = 3). One-way ANOVA, Newman–Keul’s post-test. ^∗^*P* < 0.05, ^∗∗^*P* < 0.01, ^∗∗∗^*P* < 0.001. Corresponding cumulative probability plots are shown at the right. **(G,H)** Representative images of AMPAR immunoreactivity in the control and chronically epileptic hippocampus, respectively. Left panel, overview; right panel, high-magnification confocal image. iml, inner molecular layer; mml, middle molecular layer; oml, outer molecular layer; slm, stratum lacunosum moleculare. **(I)** Quantitative analysis of AMPAR optical density in the molecular layer. Number of mice: *n*_cntrl_ = 4; *n*_KA,contra_ = 4, *n*_KA,ipsi_ = 4. One-way ANOVA, Newman–Keul’s post-test. ^∗^*P* < 0.05, ^∗∗^*P* < 0.01, ^∗∗∗^*P* < 0.001.

Given that *Arc* mRNA translocation to the dendritic compartment (as a molecular readout for strong excitatory input) was not significantly changed, despite during SE, we asked whether excitatory post-synaptic contact sites (i.e., dendritic spines) were altered on the structural or molecular level (**Figure 5**; *n*_cntrl_ = 4; *n*_SE_ = 3, *n*_1w_ = 4, *n*_2w_ = 3, *n*_3w_ = 5).

In the sclerotic, ipsilateral septal hippocampus, we observed a pronounced increase of spine density on eGFP-positive DGC dendrites crossing the termination zone of entorhinal afferents. This increase was evident 1 week after SE and persisted during the following 2 weeks of epileptogenesis (**Figures 5A, B2**; control: 0.43 ± 0.03/μm; SE: 0.44 ± 0.03/μm; 1w: 0.65 ± 0.05/μm, *P* < 0.01; 2w: 0.58 ± 0.03/μm; *P* < 0.05; 3w: 0.60 ± 0.02/μm, *P* < 0.01). Conversely, in non-sclerotic regions (contralateral septal and ipsilateral temporal) the spine density did not change significantly, but displayed a trend toward a decrease of spine numbers on contralateral DGCs 3 weeks after KA (**Figure 5B1**; control: 0.47 ± 0.03/μm, 3w: 0.34 ± 0.05/μm, *P* = 0.055). Moreover, ultrastructural analysis revealed that DGC spines in both sclerotic and non-sclerotic regions show remarkable changes in synaptic fine structure, i.e., increase of spine perimeter, PSD length and number (**Figures 5C–F**; *n*_cntrl_ = 3, *n*_contra-sept_ = 5; *n*_ipsi-sept_ = 6; *n*_ipsi-temp_ = 6). DGC spines in the ipsilateral septal molecular layer exhibited the strongest alterations (spine perimeter, control: 1.03 ± 0.02 μm; KA, ipsi sept: 2.40 ± 0.10 μm, *P* < 0.001; PSD length per spine, control: 143.3 ± 10.74 nm, KA, ipsi sept: 390.1 ± 13.75 nm, *P* < 0.001; number of PSDs per spine, control: 1.03 ± 0.03; KA, ipsi sept: 1.80 ± 0.10, *P* < 0.001), but also spines on the contralateral septal and ipsilateral temporal side showed a significant enlargement of the perimeter (KA, contra sept: 1.91 ± 0.04 μm, *P* < 0.001; KA, ipsi temp: 2.13 ± 0.07 μm, *P* < 0.001) and an increase of the summed PSD length per spine (KA, contra sept: 264.0 ± 11.14 nm, *P* < 0.001; KA, ipsi temp: 312.30 ± 6.55 nm, *P* < 0.001). Interestingly, although the increase of spine size and density would have predicted an elevation of post-synaptic glutamate receptors (Nusser et al., 1998; Takumi et al., 1999; Racca et al., 2000), we found a highly significant decrease of AMPARs by about 50% in the ipsilateral septal molecular layer (**Figures 5G–I**; optical density, control: 53.9 ± 0.7; KA, contra: 50.9 ± 2.4; KA, ipsi: 27.6 ± 2.4, *P* < 0.001), the same region where DGCs displayed (i) elevated *Arc* mRNA levels and (ii) showed the most prominent increase in spine number and size. A similar asymmetric relationship between *Arc* increase and AMPAR decrease was also evident in both hippocampi during SE (**Supplementary Figure 6**), suggesting that both factors are mechanistically linked also under epileptic conditions.

In summary, we show that in the sclerotic hippocampus DGC spines in the target zone of entorhinal afferents display morphological and molecular adaptations that are considered to oppose each other on the functional level: The multiplication and enlargement of spines indicate an increase of synaptic strength, whereas the accompanied reduction of AMPAR expression, predicts a weakening of synaptic efficacy.

### Seizure-Induced Responses Are Altered in Dispersed DGCs

**FIGURE 6:**
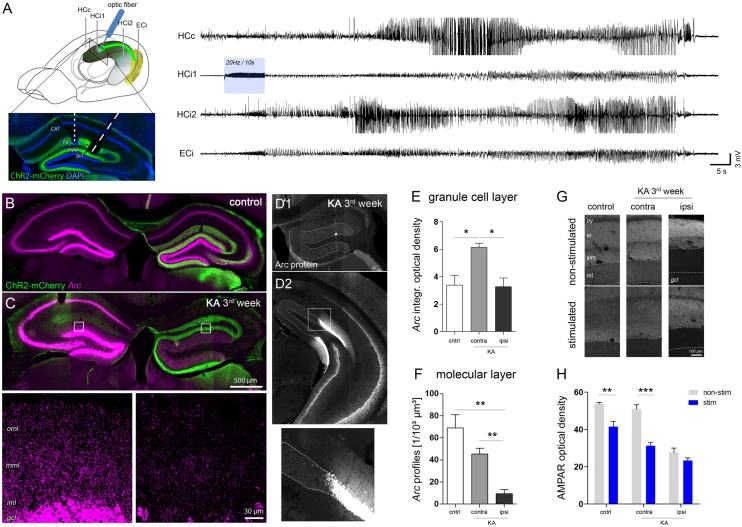
Region-specific responses of DGCs to optogenetically-induced seizures. **(A)** Schematic showing the position of electrodes (HCc, contralateral hippocampus; HCi1, ipsilateral septal; HCi2, ipsilateral temporal; ECi, ipsilateral entorhinal cortex). Brain sketch modified from Witter and Amaral (2004). Optic fibers were implanted in the ipsilateral septal region to obtain a spatially confined stimulation of ChR2-expressing entorhinal afferents (ChR2-mCherry, green). Representative LFP recording on the right shows an optogenetically-evoked generalized seizure. Blue shading denotes the local delivery of pulsed blue light, here, at 20 Hz for 10 s. **(B,C)** Representative images showing seizure-induced upregulation of *Arc* mRNA (magenta) in the hippocampus of healthy controls and chronically epileptic mice, respectively. High-magnification confocal images for **C** are shown below. Note the lack of *Arc* mRNA upregulation in DGCs of the ipsilateral, sclerotic hippocampus. **(D1–D2)** Representative image of immunohistochemical staining for Arc protein. **(E)** Corresponding quantification of t *Arc* mRNA density in the granule cell layer and **(F)** the molecular layer. Number of mice: *n*_cntrl_ = 4, *n*_KA-contra_ = 3, *n*_KA-ipsi_ = 4. One-way ANOVA, Newman–Keul’s post-test. ^∗^*P* < 0.05, ^∗∗^*P* < 0.01. **(G)** Comparison of AMPAR density in stimulated mice which experienced a series of evoked behavioral seizures and in non-stimulated mice. **(H)** Corresponding quantitative analysis of AMPAR density in the molecular layer of the hippocampus of stimulated (blue bars) and non-stimulated (gray bars) controls (*n*_non-stim_ = 4; *n*_stim_ = 4) and chronically epileptic mice (*n*_non-stim_ = 4; *n*_stim_ = 4). Two-way ANOVA, Bonferroni’s post-test. ^∗∗^*P* < 0.01, ^∗∗∗^*P* < 0.001.

In order to elucidate the response of DGCs in the chronic epileptic hippocampus to excessive input on altered synapses, we chose an optogenetic approach in which we induced generalized, behavioral seizures via local stimulation specifically of entorhinal afferents (**Figure 6A**). Again, we investigated *Arc* mRNA expression to identify strong synaptic input and highly active neurons.

In both, KA-injected chronically epileptic mice (*n* = 4) and non-epileptic controls (*n* = 4) we induced 5–10 behavioral seizures by applying pulsed light for 10 s at 10–20 Hz to ChR2-expressing entorhinal afferents in the ipsilateral septal hippocampus. Similar to the *Arc* mRNA pattern during SE, we found a strong seizure-induced upregulation of *Arc* mRNA in all DGCs, pyramidal cells and hilar interneurons in both hippocampi of control mice (**Figure 6B**). KA-injected mice, however, displayed a clearly asymmetric expression pattern with an apparently lower *Arc* expression in remaining DGCs in the sclerotic hippocampus (**Figure 6C**). In fact, quantitative analysis demonstrated that the density of somatic *Arc* mRNA in these cells was significantly lower compared to DGCs on the contralateral side (KA, contra: 6.15 ± 0.28; KA, ipsi: 3.27 ± 0.64, *P* < 0.05), but not different from DGCs of stimulated controls, irrespective of the side (**Figure 6E**, control: 3.40 ± 0.70). In contrast, the density of dendritic *Arc* mRNA was also significantly decreased compared to stimulated controls (**Figure 6F**, control: 69.04 ± 11.89/10^3^ μm^3^, KA, contra: 45.41 ± 5.20/10^3^ μm^3^, KA, ipsi: 9.25 ± 4.01/10^3^ μm^3^, both *P* < 0.01). Intriguingly, this impairment of seizure-induced *Arc* mRNA upregulation was highly restricted to dispersed DGCs in the sclerotic hippocampus, displaying a sharp recovery with transition to non-dispersed/non-sclerotic regions. The same pattern was precisely paralleled by Arc protein expression (**Figure 6D**). Moreover, as a putative functional consequence of seizure-induced *Arc* upregulation in non-dispersed DGCs, we found a significant decrease of the AMPAR density in the molecular layer compared to non-stimulated controls (**Figures 6G,H**; control, no-stim: 53.90 ± 0.68, stim: 41.53 ± 2.84, *P* < 0.01; KA, no-stim: 50.88 ± 2.44, stim: 31.34 ± 1.62, *P* < 0.001). In contrast, no further decrease of AMPAR was observed in the ipsilateral dispersed dentate gyrus upon seizure induction (no-stim: 27.60 ± 2.43; stim: 23.30 ± 1.48).

In conclusion, our results suggest that seizure-induced translocation of *Arc* mRNA and subsequent downregulation of AMPAR in activated DGC dendrites is compromised in sclerotic regions of the hippocampus.

### Seizure Susceptibility Upon Entorhinal Input Is Increased in the Sclerotic Hippocampus

From the present results (i.e., enlargement and *de novo* formation of spines, but impaired *Arc* translocation and decreased AMPAR density) it does not become clear, how DGCs in the sclerotic region are prone to generate seizures upon synaptic input. Therefore, we determined the probability to evoke epileptic activity by optogenetic stimulation of entorhinal afferents in both, healthy controls (*n*_cntrl_ = 5) and KA-injected epileptic mice (*n*_epi_ = 8).

**FIGURE 7:**
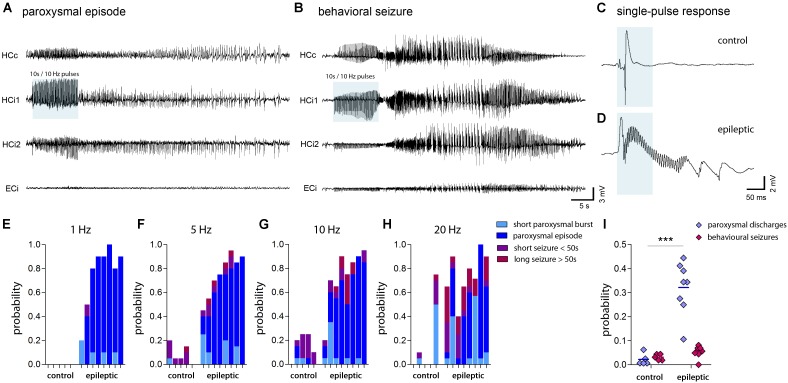
Seizure probability probed by optogenetic activation of entorhinal input. **(A,B)** Representative LFP recordings at multiple locations (HCc, contralateral hippocampus; HCi1, ipsilateral septal; HCi2, ipsilateral temporal; ECi, ipsilateral entorhinal cortex) during optogenetically-induced subclinical paroxysmal episodes or behavioral seizures. Blue shading denotes the local delivery of pulsed blue light to ChR2-expressing entorhinal afferents. **(C,D)** LFP responses evoked by a single light pulse of 50 ms (blue shading) in the ipsilateral hippocampus of healthy controls and chronically epileptic mice, respectively. Note the induction of fast ripple-like oscillations. **(E–H)** Histograms showing the probability to induce different forms of epileptic activity (short bursts, light blue; subclinical paroxysmal episodes, dark blue; short behavioral seizures, violet; long behavioral seizures, magenta) at various stimulation frequencies (1, 5, 10, and 20 Hz). Each bar corresponds to one mouse. **(I)** Quantitative analysis of the overall probability to induce either subclinical epileptiform activity (short bursts and longer paroxysmal episodes) or behavioral seizures (short and long seizures) averaged for different stimulation frequencies. Each data point corresponds to one mouse (*n*_cntrl_ = 5, *n*_epi_ = 8). Two-way ANOVA, Bonferroni’s post-te*s*t, ^∗∗∗^*P* < 0.001.

Local stimulation of entorhinal afferents in the ipsilateral septal hippocampus induced either short paroxysmal bursts consisting of about 5–10 high-amplitude spikes, longer paroxysmal episodes (>20 s of continuous spiking), short (<50 s), or long (>50 s) behavioral seizures. Taking into account the restricted penetration depth of 473 nm light in brain tissue (Yizhar et al., 2011) and an output of 60 mW/mm^2^ at the tip of our optic fiber, we estimated that in our case the radius of light emission with sufficient power to activate ChR2 does not exceed 250 μm. Apparently, locally-induced activity propagated also to the contralateral and the ipsilateral temporal hippocampus in both, control and epileptic mice (**Figures 7A,B**). We also observed induced population spikes in the entorhinal cortex, however, it is not clear whether this is due to the generation of antidromic action potentials in entorhinal afferents, or actually refers to a feed-forward propagation of signals within the hippocampal-entorhinal loop. Remarkably, in the dentate gyrus single light-induced LFP responses displayed substantial differences between control and epileptic mice: Under control conditions evoked potentials were transient and biphasic with a negative potential preceding the positive spike (**Figure 7C**). In epileptic mice, however, the negative component was strongly diminished and high-frequency oscillations emerged superimposing a slowly decaying positive wave (**Figure 7D**). No-virus control mice displayed no LFP responses upon stimulation (data not shown). Importantly, upon stimulation with pulsed light at 1, 5, 10, or 20 Hz for 10 s epileptic mice had a significant higher probability to generate subclinical paroxysmal discharges (**Figure 7I**; control: 2.5 ± 1.3%, epileptic: 34.4 ± 2.8%, *P* < 0.001). Interestingly, the pattern of induced paroxysmal episodes resembled the pattern of spontaneous paroxysmal episodes, which was stable within, but variable across KA-injected mice (**Supplementary Figures 7A–C**). Epileptic mice also showed a strong trend towards a higher probability for induced behavioral seizures (**Figure 7I**; control: 3.1 ± 0.6%; epileptic: 5.3 ± 0.9), which appeared to depend on the spatial extent of the spontaneously epileptic network (**Supplementary Figure 7D**). In controls the probability to induce a behavioral seizure peaked at 5–10 Hz stimulation, whereas in epileptic animals seizures were longer and more severe, and increased with the stimulation frequency (**Figures 7E–H**), suggesting that in the sclerotic hippocampus the gating or filtering of afferent input to DGCs is impeded.

## Discussion

In the present study, we characterized the spatiotemporal properties of hippocampal epileptic activity during epileptogenesis in mice, and determined the corresponding changes of synaptic structure and neuronal responses upon synaptic input. We focused mainly on DGCs, since these neurons together with CA2 pyramidal cells represent the major cell population surviving excitotoxic injury during SE (Häussler et al., 2016) and thus, are preserved in the sclerotic hippocampus. We report remarkable differences regarding the structural and molecular plasticity of DGCs residing in sclerotic and non-sclerotic regions of the epileptic hippocampus. Importantly, although paroxysmal discharges are present in the whole hippocampus, differences in discharge amplitudes are associated with specific patterns of activity-dependent gene expression and synaptic plasticity of DGCs. Thus, our results shed light on the structure-function relationship in the hippocampus during the development of mTLE. We would like to point out that the presented data mainly allows to estimate the changes of seizure characteristics, *Arc* mRNA expression levels and spine densities (and their relation to each other) over the time course of epileptogenesis, and to compare these changes in the sclerotic versus non-sclerotic hippocampus.

### Spatiotemporal Pattern of Hippocampal Seizure Activity

The intrahippocampal KA mouse model of mTLE recapitulates the major pathological hallmarks of the human disease, comprising unilateral hippocampal sclerosis and the emergence of spontaneous recurrent seizures (Bouilleret et al., 1999). Although the existence of a latent, seizure-free period has been reported for the present model (Riban et al., 2002; Arabadzisz et al., 2005; Janz et al., 2017b), a detailed quantitative description of the development of specific seizure characteristics comparing sclerotic with non-sclerotic regions is missing.

Our data shows, that on both sides of the hippocampus subclinical paroxysmal bursts are present early after SE and persist during 3 weeks of epileptogenesis. Notably, in the first 2 weeks after SE – typically considered as the latent phase – paroxysmal bursts in the sclerotic hippocampus display very low amplitudes and therefore do not represent seizure-like events as observed in the chronic stage of mTLE (Janz et al., 2017b). In the present study we used an algorithm customized to detect also these low-amplitude bursts and to infer their specific features, in both sclerotic and non-sclerotic regions of the epileptic hippocampus. Our data clearly implicates that the latent period is not “silent” (also shown by Heinrich et al., 2011), but one might consider, that only during this period the sclerotic hippocampus is free of high-amplitude recurrent seizures. Whether this phase is also free of behavioral seizures cannot be answered in the present study, since in the intrahippocampal KA mouse model behavioral seizures are rare and sporadic video-EEG recordings is prone to miss these seizures. Conversely, several studies applying long-term, continuous video-EEG recording in systemic chemoconvulsant or perforant path kindling MTLE models detected behavioral seizures within the first days after SE, emphasizing that epileptogenesis can be immediate and requires no secondary mechanisms (reviewed in Sloviter, 2008). However, another study, which has thoroughly investigated the emergence of seizures in systemically KA-injected rats, showed that the time between SE and the onset of subclinical focal seizures is about 1 week, whereas it is about 2 weeks for behavioral seizure onset (Williams et al., 2009). These conflicting results may indicate that the period until seizure onset highly depends on the applied animal model and experimental parameters, e.g., the strain or the severity of brain injury. Moreover, Williams et al. (2009) showed that the increase in seizure frequency over time follows a sigmoidal but not a step function, suggesting that epileptogenesis represents a gradual process, which progresses far beyond the first clinical manifestation. Considering that the temporal evolution of epileptic activity varies in different regions of the hippocampal formation, the concept of epileptogenesis becomes even more complicated. In fact, our results show that mainly the amplitude of paroxysmal discharges is modulated in a position- and time-dependent manner. Accordingly, we found that the amplitudes are generally higher in non-sclerotic regions, but that in the sclerotic hippocampus the power of paroxysmal bursts increase gradually over time. These amplitude differences may reflect the anatomical and functional properties of the intact versus the disrupted network due to excitotoxic injury. Accordingly, lower amplitudes in the acutely KA-injected hippocampus can be explained by an initial depolarization block secondary to direct actions of KA (Le Duigou et al., 2005), which is followed by progressive cell loss during subsequent epileptogenesis decreasing the number of cells participating in LFP generation. In turn, the gradual increase of amplitudes might be a result of increased input onto newly-generated DGC spines or partial recovery from neuronal dysfunction. Our results are in line with Häussler et al. (2012), showing that the power of epileptiform activity depends on the septotemporal position in the hippocampal network, i.e., being higher near the transition to non-sclerotic regions where proliferation of hyperexcitable juvenile neurons recovers. We expand this view by providing evidence that in the temporal region adjacent to the transition zone the mean burst duration is also higher, whereas the spike rate within bursts is lower. Moreover, we show that both parameters gradually decrease during disease progression, suggesting that in the sclerotic hippocampus also compensatory mechanisms may evolve over time. However, it needs to be considered that epileptogenesis in the intrahippocampal KA model could be very different to epileptogenesis in human mTLE, and also to epileptogenesis in systemic animal models for mTLE (i.e., intraperitoneal pilocarpine injection or hyperthermia), in which hippocampal sclerosis is minimal and seizures do not necessarily emerge from the hippocampus. In this context, our findings may be translated mainly to human mTLE including hippocampal sclerosis with an epileptic focus confined to the hippocampus.

In order to reveal neuronal circuits contributing to the observed changes in hippocampal seizure generation, we detected *Arc* mRNA on the sub-cellular level using FISH. The *Arc* gene, belonging to the family of immediate early genes, is transcribed within minutes upon neuronal activity mediated by NMDAR and voltage-gates calcium channels activation, however, also a slower component via metabotropic glutamate receptors exists in which the mRNA translation precedes its transcription (Shepherd and Bear, 2011). Once expressed, the *Arc* mRNA has a half-life of 47 min (Rao et al., 2006) and is actively transported along the dendrite with an average speed of about 15 μm/min and a maximum speed of 60 μm/min (Dynes and Steward, 2007). The *Arc* mRNA accumulates at postsynaptic spines receiving strong, chronic input, where it is then locally translated to mediate different forms of synaptic plasticity (Steward et al., 1998, 2015). Hence, in our study the detection of *Arc* mRNA served both, to identify active cell populations and to estimate their relative synaptic input. Accordingly, we show that full-blown seizures during SE and optogenetic stimulation extensively upregulate *Arc* expression in pyramidal cells, DGCs and some hilar neurons throughout the hippocampal formation, suggesting that these cells are recruited to the seizing network. Interestingly, only few putative GABAergic interneurons (according to their location and sparse distribution) seem to upregulate *Arc* mRNA although these cells are most likely activated downstream by pyramidal cells and DGCs, indicating that *Arc* mRNA predominantly labels active excitatory neurons. In these neurons the expression level and sub-cellular localization of *Arc* mRNA appear to directly reflect the strength of seizure activity, since *Arc* mRNA is highly expressed and transported to DGC dendrites in regions exhibiting high amplitudes of paroxysmal discharges (i.e., in the non-sclerotic hippocampus during SE). Intriguingly, this expression pattern is reversed after SE: During 3 weeks of epileptogenesis, enhanced *Arc* expression is restricted to dispersing DGCs near the KA injection site, suggesting that these cells are persistently hyperactive. Corroborating the idea that *Arc* mRNA levels indeed reflect the history of DGC activity, three major observations can be pointed out: The upregulation of *Arc* mRNA (1) is positively correlated with the burst ratio, (2) follows the increase of burst amplitudes and (3) is augmented by longer paroxysmal episodes. Although under epileptic conditions the input-output relation of individual DGCs *in vivo* remains elusive, it is well conceivable that in the sclerotic hippocampus recurrent excitation, due to mossy fiber sprouting (Buckmaster et al., 2002) and local disinhibition, caused by the loss of GABAergic interneurons (Marx et al., 2013) and their synapses with DGCs (see supplements of Janz et al., 2017a) elevate the activity of DGCs. It is known that these pro-epileptic changes are accompanied also by compensatory changes of DGCs that attenuate their intrinsic excitability by decreasing calbindin-dependent calcium influx (Nägerl et al., 2000) and increasing potassium outflux mediated by inward rectifier potassium channels (Young et al., 2009; Stegen et al., 2012). However, intrinsic rescaling of DGCs might not prevent hyperactivity, but actually restore specific functions of the dentate network under epileptic conditions e.g., pattern separation (Yim et al., 2015). The picture emerges that during epileptogenesis hyperactive DGCs residing in the sclerotic hippocampus generate paroxysmal activity (Pallud et al., 2011) which then propagates to other non-sclerotic parts of the hippocampal formation. Taking into account the known anatomy of the sclerotic hippocampus, paroxysmal discharges may propagate via two distinct pathways: (1) Through recurrent mossy fiber connections to temporal DGCs and consequently to the locally intact hippocampal-entorhinal pathways, and (2) through remaining CA3 and CA2 pyramidal cells forwarding the signals to the contralateral and ipsilateral temporal CA1 pyramidal cells (Shinohara et al., 2012) as well as extra-hippocampal regions (Dudek et al., 2016). In fact, the propagation of optogenetically-evoked discharges to the contralateral and temporal hippocampus verified the presence of both, commissural and septotemporal pathways in the sclerotic part.

Beyond the hippocampus, seizures were shown to reverberate within the entorhinal-hippocampal loop in isolated guinea pig brains (Uva et al., 2005; Boido et al., 2014), but recent *in vivo* studies found a feed-forward propagation of acute seizures from the hippocampus to the entorhinal cortex, rather than a re-entrant to the hippocampus (Lu et al., 2016). This is remarkable, because DGCs receive the majority of their excitatory input from the entorhinal cortex via the perforant path and this projection is even augmented in the sclerotic hippocampus of chronically epileptic mice (Janz et al., 2017a). However, in this region *Arc* mRNA is only upregulated in DGC somata but not in the dendritic compartment, neither during SE nor during chronic epilepsy. This suggests that functional perforant path input is either not sufficient to drive translocation, or that *Arc* mRNA translocation in DGCs of the sclerotic hippocampus is impaired *per se*, considering that the entorhinal cortex appears largely preserved in this model (Janz et al., 2017a). Accordingly, the lack of *Arc* mRNA translocation in the sclerotic hippocampus could be explained either by a local interruption of the hippocampal-entorhinal loop due to an initial depolarization block (Le Duigou et al., 2005), a later loss of hippocampal efferents to the entorhinal cortex (Janz et al., 2017a), or by an Arc/AMPAR-dependent homeostatic scaling of individual DGC synapses, as discussed below.

### Synaptic Plasticity and Altered Responses to Excitatory Afferent Input

Several lines of evidence suggest that synaptic Arc regulates both, hebbian forms of plasticity and non-hebbian, homeostatic scaling of postsynaptic efficacy (Bramham et al., 2010; Korb and Finkbeiner, 2011; Shepherd and Bear, 2011; Steward et al., 2015). In line with the role of Arc in spine formation (Peebles et al., 2010), we show that in DGCs of the sclerotic hippocampus upregulation of *Arc* expression is followed by an increase of the spine density 1 week after SE. Previously, we showed that these newly-formed spines receive increased excitatory input by the medial entorhinal cortex (Janz et al., 2017a), which might contribute to DGC hyperexcitation in mTLE. Our present data suggest that, reorganization of entorhinal input to DGCs is concluded during the first week of epileptogenesis, which is relatively fast compared to the formation of recurrent mossy fiber synapses starting between the first and the second week after SE (Janz et al., 2017b). Although the DGC spine density and the size of individual spines are augmented in the sclerotic hippocampus, *Arc* mRNA does not translocate to these synapses upon optogenetic activation, which indicates a remodeling of synaptic function. Consistent with this interpretation, we show that the overall AMPAR density is strongly attenuated in the molecular layer, predicting a decrease of postsynaptic efficacy and NMDAR-dependent *Arc* expression. Indeed, *Arc* upregulation caused by excessive synaptic input is a well described regulatory mechanism that could mediate a decrease of AMPAR in DGCs (Rial Verde et al., 2006; Shepherd et al., 2006; Korb et al., 2013). However, it is important to note that different AMPAR isoforms were shown to be contrarily regulated under epileptic condition (Suzuki et al., 2000), so the netto effect on synaptic efficacy remains unclear. It is also not clear how much the loss of interneurons contributes to the decrease in AMPAR density. Conversely, in the non-sclerotic hippocampus both, the spine and AMPAR densities are not affected, the *Arc* mRNA levels are similar to control conditions, and the seizure-induced *Arc* upregulation is preserved. These results demonstrate that structural and functional plasticity of DGCs in the epileptic hippocampus is regulated in a highly position-dependent fashion. Although the causal relationship between position-dependent synaptic plasticity and local seizure activity remains to be determined, we hypothesize that low-power activity at the KA injection site, triggers Arc-dependent synaptic scaling and formation of new synapses to restore the excitation level of DGCs, consistent with the idea of homeostatic structural plasticity (Fauth and Tetzlaff, 2016). In turn, excessive synapses on DGCs might destabilize the local network when activated in a non-physiological manner. In fact, we show that in the heavily sclerotic hippocampus of epileptic mice, optogenetic stimulation of entorhinal synapses evoked robust epileptic activity, which was not pronounced in healthy animals. Previous studies reported that in healthy rats the probability to induce full-blown seizures by optogenetic stimulation of ChR2-expressing DGCs depends on the light-pulse frequency (Osawa et al., 2013). Our results corroborate this finding, but also reveal that the probability to induce subclinical paroxysmal discharges is similar over a broad frequency range (1–20 Hz) and that these seizure-like episodes are virtually exclusive for epileptic mice. In line with our observations, there is recent evidence that the gating function of DGCs is compromised under epileptic conditions (Krook-Magnuson et al., 2013, 2015). While formation of new aberrant entorhinal synapses on DGC likely contribute to this phenomenon (Janz et al., 2017a), other pathological changes are clearly involved in aggravating DGC excitability: (i) Loss of inhibitory interneurons (Marx et al., 2013; Hofmann et al., 2016) and mossy cells (Bui et al., 2018), (ii) dysfunction of astrocytes (Steinhäuser et al., 2012; Bedner et al., 2015; Albright et al., 2016) and (iii) pathological expression of KA receptors on sprouted mossy fibers (Artinian et al., 2011, 2015; Peret et al., 2014).

## Conclusion

Taken together, our study shows that during the development of mTLE, position- and time-dependent changes of hippocampal seizure activity are associated with distinct alterations of the structure and function of DGC synapses, particularly involving regions of the hippocampus which become sclerotic during disease progression. Here, *Arc* emerges as an important molecule that could link activity changes to synaptic reorganization, and signifies DGCs as a hyperactive cell population in the sclerotic hippocampus. However, one has to bear in mind that the sclerotic region might not generate full-blown seizures independently, but may rely on the adjacent, non-sclerotic networks. Therefore, future studies will have to further differentiate epileptogenic from compensatory synaptic alterations in a region- and time-specific manner, and to elucidate how different hippocampal sub-regions interact to promote seizure activity.

## Author Contributions

PJ designed and performed the experiments, analyzed the data, and wrote the manuscript. PH performed the experiments, analyzed the data, and contributed to the manuscript. KH provided the algorithm for electrophysiological data analysis, helped with the data analysis, and contributed to the manuscript. SN acquired all electron microscopy images. MK provided the resources for electron microscopy. UE critically revised the manuscript and added intellectual content. CH conceptualized the study and helped writing the manuscript.

## Conflict of Interest Statement

The authors declare that the research was conducted in the absence of any commercial or financial relationships that could be construed as a potential conflict of interest.
