# Supplementary material for: Position- and Time-Dependent Arc Expression Links Neuronal Activity to Synaptic Plasticity During Epileptogenesis
Source: Front Cell Neurosci. 2018 Aug 14;12:244. doi: 10.3389/fncel.2018.00244 (PMC6102356; doi:10.3389/fncel.2018.00244)
Supplement: Supplementary file 1 [file Data_Sheet_1.pdf]

Supplementary figures

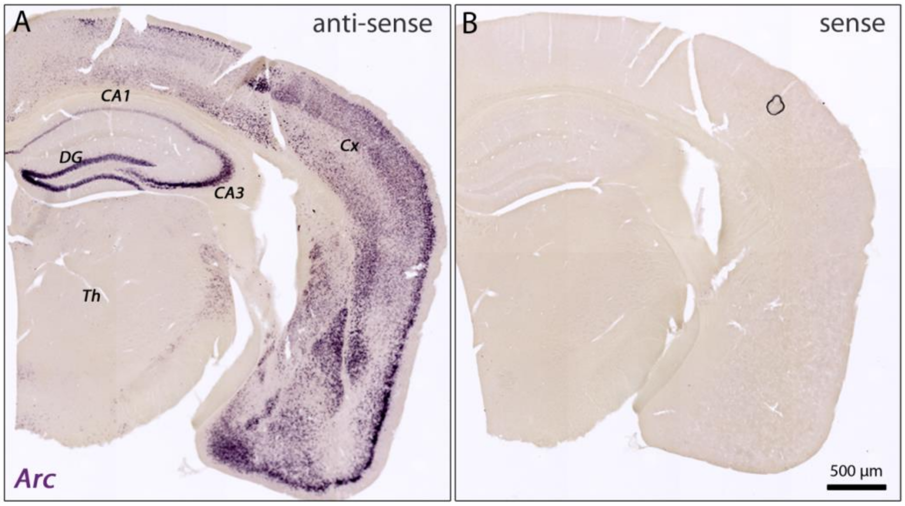

Supplementary figure 1: Specificity of *Arc* mRNA detection. (A) Colorimetric detection of *Arc* mRNA using the anti-sense probe. The brain slice was taken from a mouse during SE. CA, cornu ammonis; DG, dentate gyrus; Cx, cortex; Th, Thalamus. (B) No staining is observed following hybridization with the sense probe.

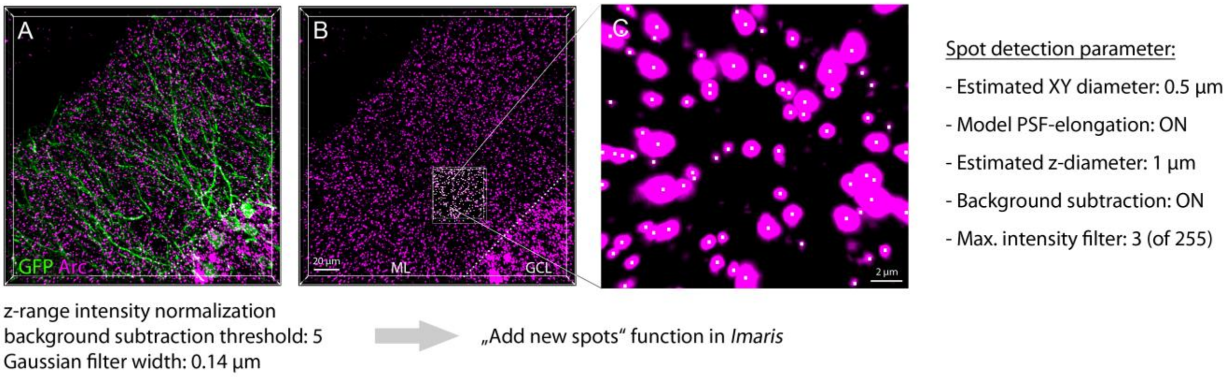

Supplementary figure 2: Automated 3D-detection of *Arc* mRNA profiles. (A) 3D representation of a confocal z-stack. Granule cells are intrinsically labeled with eGFP (green). *Arc* mRNA was detected using FISH (magenta). Post-processing parameters are depicted below. (B) In a representative ROI (40 x 40 x 20 µm³), individual *Arc* mRNA profiles were automatically detected using *Imaris*. (C) Zoomed image shows the sparse distribution of *Arc* mRNA profiles in the molecular layer (ML). Signal intensity was saturated to improve the visibility of *Arc* mRNA profiles with low intensity. Automatically detected profiles are marked with white squares. Detection parameters are shown on the right.

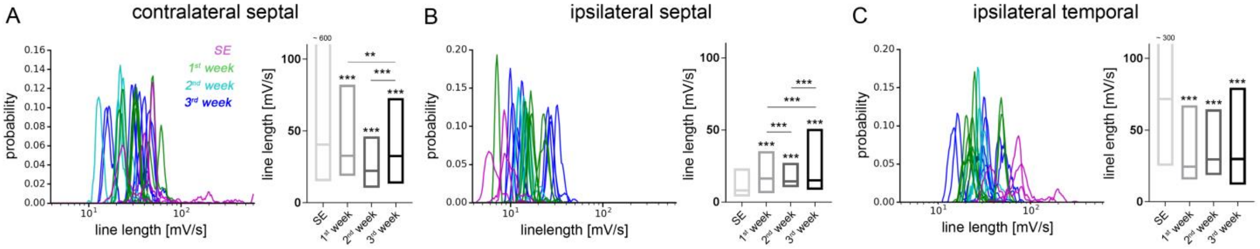

Supplementary figure 3: Region-dependent changes in epileptic burst line length. The line length of epileptic episodes (i.e. voltage change of the burst) normalized to their duration was analyzed for different hippocampal regions: (A) contralateral septal, (B) ipsilateral septal and (C) ipsilateral temporal. Right panels display the probability plots. Plotted lines represent data from individual animals. Number of mice: n_{SE} = 3 (magenta), n_{1w} = 4 (green), n_{2w} = 3 (light blue), n_{3w} = 5 (dark blue). Left panels display box and whisker plots. Data displayed as median and min to max. Tested data points correspond to individual bursts. One-way ANOVA, Newman-Keul's post-test. ** P < 0.01, *** P < 0.001.

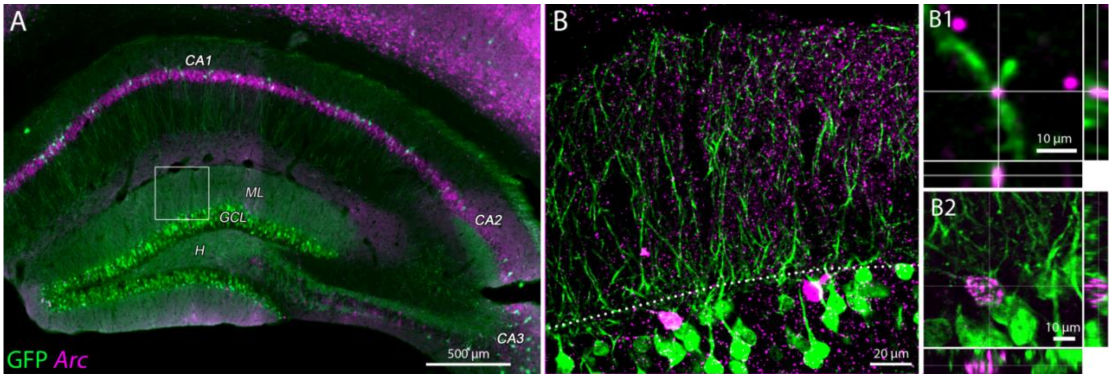

Supplementary figure 4: FISH-based localization of *Arc* mRNA under control conditions. (A) Representative image of a saline-injected hippocampus (control conditions) identifying *Arc* mRNA expression (magenta) mainly in pyramidal cells of CA1. A subpopulation of CA1 pyramidal cells and DGCs are labeled with endogenous GFP (green). H, hilus; GCL, granule cell layer, ML, molecular layer. (B) High-magnification confocal image of the dentate gyrus reveals sparse *Arc* expression in somata and dendrites of DGCs. (B1-2) Orthogonal views of *Arc* mRNA profiles localized at the base of a spine or in the nucleus of DGCs.

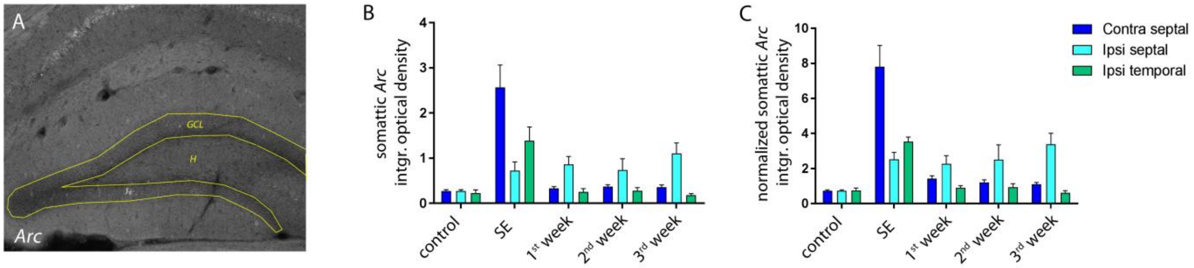

Supplementary figure 5: Quantification of somatic *Arc* mRNA with and without normalization. (A) Representative confocal image showing *Arc* mRNA labeled with FISH. Image was converted to grey-scale. The integrated optical density of *Arc* mRNA labeling was measured in the granule cell layer (GCL). The ROI is illustrated with yellow outlines. For background normalization, the signal intensity in the hilus (H) was taken as a reference. (B) *Arc* mRNA optical density values without and (C) with normalization. Number of mice: n_{cntrl} = 3-5, n_{SE} = 3, n_{1w} = 3, n_{2w} = 3, n_{3w} = 5. Note that both analyses result in similar relative values.

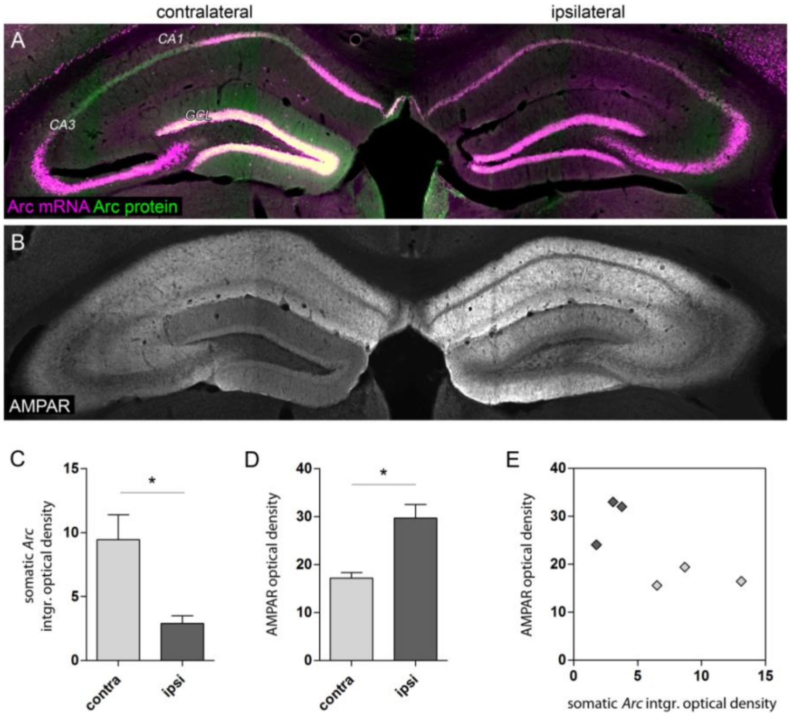

Supplementary figure 6: Relationship between *Arc* mRNA / protein and AMPAR expression during SE. (A) Representative image showing *Arc* mRNA (magenta) and Arc protein (green) expression on both sides of the hippocampus during SE. CA, cornus ammonis; GCL, granule cell layer. (B) Representative image of an adjacent section stained for AMPAR (grey). (C-D) Corresponding quantitative analysis of *Arc* mRNA and AMPAR density comparing the contralateral to the ipsilateral side during SE ($n_{contral}$ = 3, n_{ipsi} = 3, three sections per animal). Student's t-test, * $P < 0.05$. (E) Pearson's correlation between *Arc* mRNA and AMPAR density indicates an inverse relationship of both parameters during SE.

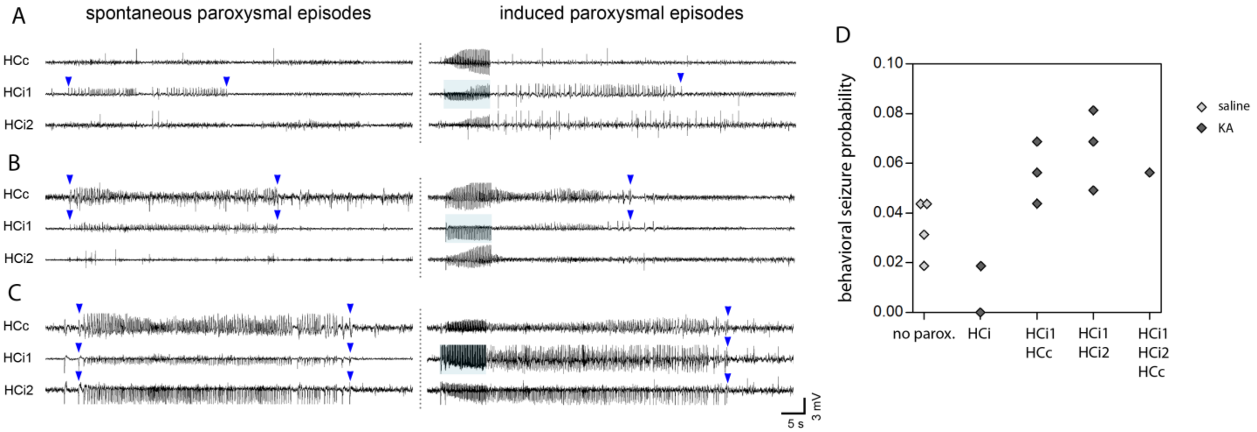

Supplementary figure 7: Relationship between the spontaneous epileptic network and seizure induction. (A-C) Representative LFP traces from three KA-injected mice showing spontaneous (left panel) and optogenetically-induced (right panel) paroxysmal episodes. Start- and end-points of paroxysmal episodes are denoted with blue arrow heads. Each example shows a different pattern or paroxysmal activity: (A) Electrographic seizures present only in the ipsilateral septal hippocampus (HCi1), (B) in both, the ipsilateral and contralateral septal hippocampus (HCi1 and HCc) but not the ipsilateral temporal hippocampus (HCi2), and (C) in all three probes regions in the hippocampal formation (HCi1, HCi2 and HCc). (D) Scatter plot showing the relationship between the probability of optogenetically-induced behavioral seizures and the pattern of paroxysmal activity (locations in which spontaneous focal seizures were evident are plotted on the x-axis) for individual mice. Each spot represents one mouse (saline-, grey; KA-injected mice, black). Saline mice displayed no paroxysmal discharges.
